# Bioelectrical State of Bacteria Is Linked to Growth Dynamics and Response to Neurotransmitters: Perspectives for the Investigation of the Microbiota–Brain Axis

**DOI:** 10.3390/ijms241713394

**Published:** 2023-08-29

**Authors:** David Muñoz-Rodríguez, Marwane Bourqqia-Ramzi, Maria Teresa García-Esteban, Antonio Murciano-Cespedosa, Alejandro Vian, Juan Lombardo-Hernández, Pablo García-Pérez, Francisco Conejero, Álvaro Mateos González, Stefano Geuna, Celia Herrera-Rincon

**Affiliations:** 1 Biomathematics Unit, Data Analysis & Computational Tools for Biology Research Group, Department of Biodiversity, Ecology & Evolution, and Modeling, Complutense University of Madrid, 28040 Madrid, Spain; 2 Molecular Biotechnology Center, University of Turin, 10126 Turin, Italy; 3 Department of Genetics, Physiology and Microbiology, Complutense University of Madrid, 28040 Madrid, Spainalejandrov@bio.ucm.es (A.V.); 4 Neuro-Computing and Neuro-Robotics Research Group, Neural Plasticity Research Group Instituto Investigación Sanitaria Hospital Clínico San Carlos (IdISSC), Complutense University of Madrid, 28040 Madrid, Spain; 5 NYU-ECNU Institute of Mathematical Sciences, Shanghai New York University, Shanghai 200122, China; agm6@nyu.edu

**Keywords:** bioelectricity, gut microbiota–brain axis, bis-(1,3-dibutylbarbituric acid) trimethine oxonol-DiBAC, depolarization

## Abstract

Inter-cellular communication is mediated by a sum of biochemical, biophysical, and bioelectrical signals. This might occur not only between cells belonging to the same tissue and/or animal species but also between cells that are, from an evolutionary point of view, far away. The possibility that bioelectrical communication takes place between bacteria and nerve cells has opened exciting perspectives in the study of the gut microbiota–brain axis. The aim of this paper is (i) to establish a reliable method for the assessment of the bioelectrical state of two bacterial strains: *Bacillus subtilis* (*B. subtilis*) and *Limosilactobacillus reuteri* (*L. reuteri*); (ii) to monitor the bacterial bioelectrical profile throughout its growth dynamics; and (iii) to evaluate the effects of two neurotransmitters (glutamate and γ-aminobutyric acid-GABA) on the bioelectrical signature of bacteria. Our results show that membrane potential (*Vmem*) and the proliferative capacity of the population are functionally linked in *B. subtilis* in each phase of the cell cycle. Remarkably, we demonstrate that bacteria respond to neural signals by changing *Vmem* properties. Finally, we show that *Vmem* changes in response to neural stimuli are present also in a microbiota-related strain *L. reuteri*. Our proof-of-principle data reveal a new methodological approach for the better understanding of the relation between bacteria and the brain, with a special focus on gut microbiota. Likewise, this approach will open exciting perspectives in the study of the inter-cellular mechanisms which regulate the bi-directional communication between bacteria and neurons and, ultimately, for designing gut microbiota–brain axis-targeted treatments for neuropsychiatric diseases.

## 1. Introduction

Cellular communication is not only mediated by biochemical signaling but also by bioelectrical communication based on signals from ion fluxes, electric fields, and voltage gradients and triggered by ion channels and pumps [1,2,3,4,5]. The bioelectrical communication system uses the endogenous bioelectricity of cells, which is not based on the propagation of action potentials, being characterized by a relatively slow speed of information transmission. Endogenous bioelectricity is involved in numerous cellular processes [6], such as wound healing [7], regulation of neurotransmitter diffusion [8], limb regeneration [9], organ development [1], and cancer development [10]. Endogenous bioelectricity generates not only short-term responses but also long-term responses [11], and new therapeutic opportunities might arise from the investigation of the bioelectrical state of cells [12].

Endogenous bioelectricity is also very important in bacteria [13]. A pioneering study in 2015 demonstrated that there is communication between bacteria comprising a biofilm through membrane potential mediated by important ion channels such as calcium, sodium, chloride, and ionotropic glutamate receptors [3]. Interestingly, this type of communication is like that observed in mammalian neurons. Bioelectrical communication between bacteria is not only established between bacteria of the same colony or biofilm [14,15]; for example, it has been shown that *Bacillus subtilis* (*B. subtilis*) biofilms can interact with distant bacteria by modulating their motility towards the biofilm, or even with other bacteria biofilms [16]. It has even been proposed that bacteria are able to store information of the membrane potential, thus generating the possibility of bacterial memory, as has been shown to occur in neurons [17]. This would open new perspectives in the study of endogenous bioelectricity in bacteria, akin to what has already occurred in the case of neurons.

The close synergetic relationship between the trillions of bacteria populating the human body (the microbiota) and the central nervous system (CNS) is well-established (see excellent reviews in [18,19,20,21]). Studies in this area have gained a great deal of interest, especially regarding the relationship between the imbalance in gut microbial communities (dysbiosis) and the occurrence of brain diseases. Alterations in brain–microbiota interactions have been suggested to be involved in the pathogenesis of various brain–gut disorders [19,20,22], as well as in neurogenerative diseases such as Alzheimer’s disease or Parkinson´s disease [23,24,25,26]. Bacteria can communicate with the CNS through the production of specific metabolic compounds, e.g., bile acids, short-chain fatty acids (SCFAs), glutamate, γ-aminobutyric acid (GABA), dopamine (DA), norepinephrine (NE), serotonin (5-HT), and histamine. Hence, the synthesis—and subsequent release—of neurotransmitters by bacteria is one of the communication mechanisms in the gut microbiota–brain axis. In this regard, two of the main neurotransmitters of the CNS, namely, glutamate (excitatory) and GABA (inhibitory) [27,28], play a key role in this synergic system; i.e., either being part of a regulatory network as molecular effectors in CNS or being involved in pH homeostasis and energy production in bacterial cells [29,30,31]. We focused on the effects of these two neuroactive molecules on bacteria since they have similar modulatory activity in bacteria and neurons, and they are ubiquitous in both biological entities, which could be an indication of a cross-kingdom communication system [32].

Most of the examples of neuron–bacteria interaction reported in the literature refer to the effect of the microbiota on the CNS, but what happens in the opposite direction? Although it has been documented that bacteria can respond to neural stimuli, little is known about how the CNS affects microbiota homeostasis. In fact, some important questions still need to be answered: (1) To what extent does the nervous system influence the microbiota? (2) What kind of mechanisms (genetic, physiological, etc.) mediate this effect? (3) Do neurons interact with bacteria directly or through some type of intermediary?

As we start exploring these questions, a key methodological issue is how to detect the bioelectrical profile of micro-organisms. Various techniques can be used, including microelectrode-based methods, optogenetics, and fluorescent probes [13,33]. The latter has proven to be an effective tool for tracking bacterial membrane potential (*Vmem*), showing several advantages over other techniques. Fluorescent probes are simple to use, and the data obtained are easy to analyze; they provide subcellular resolution images, and they allow the assessment of moving targets. In addition, the possibility of measuring changes over long periods of time makes them suitable for longitudinal studies of dynamical systems [34]. One of the most widely used *Vmem* reporters is bis-(1,3-dibutylbarbituric acid) trimethine oxonol), DiBAC4(3) (DiBAC hereon). DiBAC is an anionic lipophilic fluorescent probe that cannot pass through the polarized inner membrane of bacterial cells because of its negative charge [35]. When the bacterial membrane depolarizes, it becomes positively charged, and DiBAC can enter. As the inner leaflet becomes more positive, more DiBAC enters, and the DiBAC fluorescent signal becomes more intense [34,36] due to the binding to positively charged intracellular proteins or to the hydrophobic regions [35].

Considering all of the above, the aim of this study was first to establish a reliable method for the assessment of the bioelectrical state of two bacterial species (*B. subtilis* and *Limosilactobacillus reuteri—L. reuteri*) using DiBAC as a fluorescent probe. Then, we aimed to adopt this method with two different goals: (i) to analyze the bioelectric profile of *B. subtilis* throughout its growth dynamics and, therefore, the physiological state in each phase of the cell cycle; and (ii) to evaluate the effects of two neurotransmitters (glutamate and GABA) on the bioelectric signature of *B. subtilis* and *L. reuteri* cells.

## 2. Results

### 2.1. The Voltage-Sensitive Fluorescent Dye DiBAC Reveals Vmem Changes in B. subtilis

**Figure 1:**
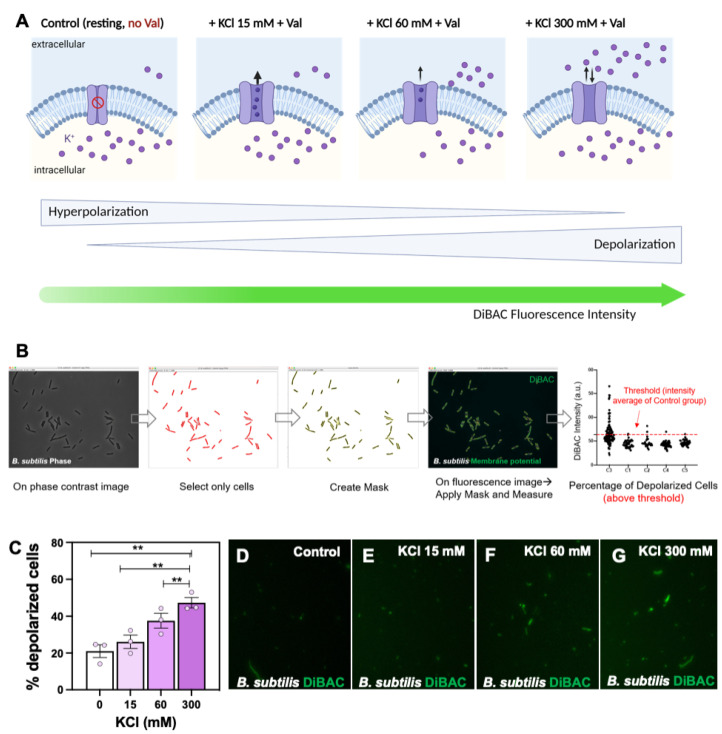
**Fluorescent voltage reporter DiBAC, image analysis and quantitative method to report membrane potential (*Vmem)* in *Bacillus subtilis (B. subtilis)*.** (**A**) Conceptual schematic showing the experimental rationale and workflow for DiBAC validation assay. Controlled increasing concentrations of potassium chloride, KCl (in presence of valinomycin, Val) are added in the extracellular medium to induce depolarization (due to K^+^ ions efflux). A chemical potassium clamp (300 mM of KCl, matching the intracellular concentration) should prevent the K^+^ gradient across the cellular membrane, reaching the maximum level of depolarization. Created with BioRender.com. (**B**) DiBAC fluorescence intensity is evaluated and quantified for each population using an ImageJ macro. The generalized estimating equations (GEE) statistical method is applied to compare the percentage of depolarization among the different treatments (KCl 0 mM, 15 mM, 60 mM, 300 mM). (**C**) Our results showed a significant increase in the percentage of depolarized cells as the KCl increases in the extracellular medium. For each experimental condition, values from three biological replicates (dots) with at least three technical replicates each are plotted. *P* values after applying GEE for percentage of depolarized cells among all groups are indicated as ** *p <* 0.01. (**D**,**G**) High-magnification images showing DiBAC-expressing *B. subtilis* cells (in green), as revealed under 40× fluorescence microscopy for Control (**D**), KCl 15 mM (**E**), KCl 60 mM (**F**), and KCl 300 mM (**G**). Scale bar = 5 µM.

Before testing for and studying the fundamental dynamics of the bioelectrical response in bacteria, a preliminary validation of the fluorescent voltage-sensitive dye Di-BAC4(3) as a reliable indicator of depolarization was necessary. To this end, we measured fluorescence levels in the presence of increasing concentrations of potassium chloride, KCl (0 mM as a Control, 15 mM, 60 mM, and 300 mM) and the K^+^-ionophore/antibiotic Valinomycin (Val 5 µM; Figure 1A). We focused on the quantification of depolarization (induced by K^+^ efflux) in individual cells within each population or sample, as our assay enables imaging at single-cell resolution, by means of establishing the thresholds for depolarization at the average of DiBAC fluorescence intensity in the Control condition (Figure 1B).

Applying generalized estimating equations (GEE), and including as factor “KCl concentration (0, 15, 60 and 300)”, we evaluated the DiBAC-expressing *B. subtilis* population in the four treatments (Figure 1C–G). Our results revealed a significant increase in the percentage of depolarized cells with increasing extracellular KCl (coefficient 0.0032, *p <* 0.001). The percentage of depolarization increased from 21.03 ± 3.48% in the Control group to 26.13 ± 3.60% in Val + KCl 15, to 37.59 ± 4.04% in Val + KCl 60, and to 47.38 ± 2.85% in Val + KCl 300, which is the expected response to the influx of K^+^ when incubation is performed in a depolarizing medium. GEE comparisons between groups showed *p <* 0.001 in all cases (see Supplementary Table S1 for statistical details; for all groups, *N* = 3 biological replicates with at least three technical replicates; n = 2808; 3243; 3438 and 3101 examined bacteria in KCl at 0; 15; 60 and 300 mM, respectively).

Taken together, our morphological data, followed by the establishment of quantitative analysis methods, demonstrated that *Vmem* changes in *B. subtilis* can be read out in real-time using DiBAC. These results validate the approach for the next experiments and the functional testing of specific hypotheses concerning bacteria’s bioelectrical state.

### 2.2. Depolarization Is Related to Increasing Proliferative Properties of Bacteria Population

**Figure 2:**
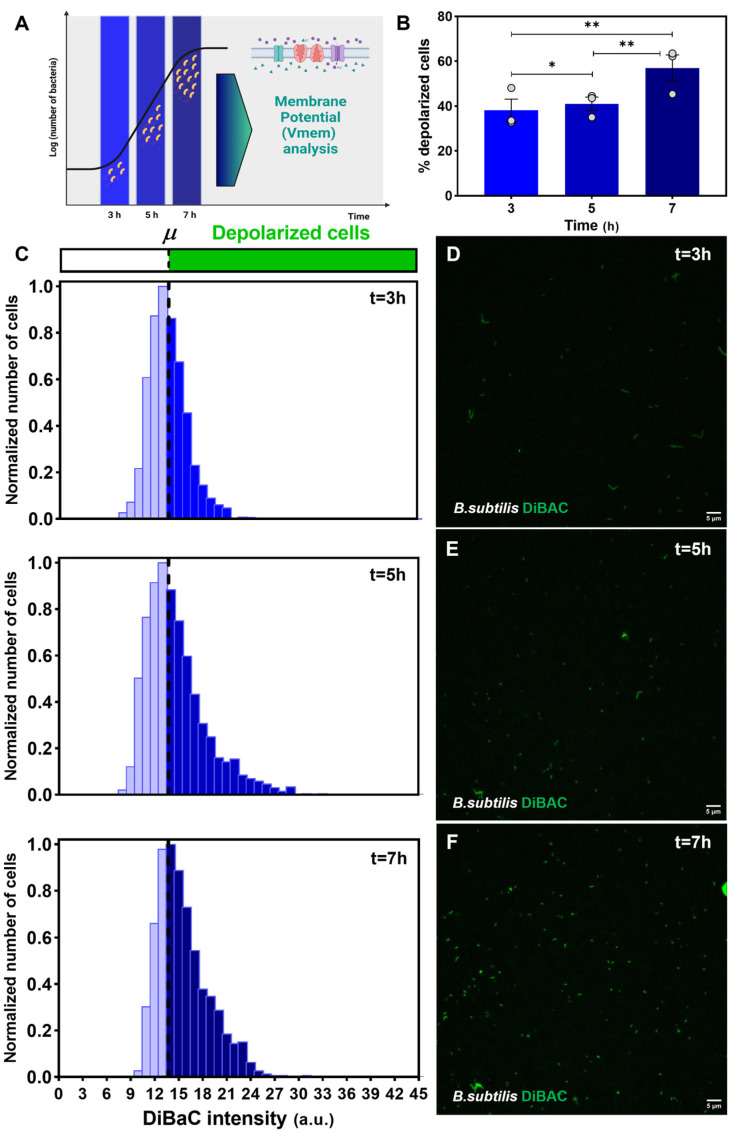
**Bioelectrical pattern throughout *Bacillus subtilis* (*B. subtilis)* growth dynamics.** (**A**) Conceptual schematic describing the basis of the experimental design for the assessment of the depolarization profile in a *B. subtilis* population. A pure culture of *B. subtilis* is inoculated in fresh medium and incubated up to >7 h. At t = 3, 5, and 7 h bacterial cells are sampled and stained with DiBAC depolarization reporter for the membrane potential (*Vmem*) analysis by epifluorescence microscopy. Created with BioRender.com. (**B**) Our results revealed a significant increase in the percentage of depolarized cells as the culture time rises. Values from three biological replicates (dots) with three technical replicates for each condition are represented per time. *p*-values after applying generalized estimating equations (GEE) statistical method for percentage of depolarized cells among all cases are indicated as * *p <* 0.05, ** *p <* 0.01. (**C**) Frequency distribution histograms of DiBAC-expressing *B. subtilis* cells according to their fluorescence intensity for t = 3 h (top), t = 5 h (middle), and t = 7 h (bottom). Data are plotted as the total number of cells, normalized to the number of those exhibiting the most frequent intensity value at each time. Depolarization threshold set at 13.702 arbitrary units (a.u; dashed line) is calculated as the average DiBAC intensity value of bacterial cells at t = 3 h. (**D**–**F**) Epifluorescence microscopy images showing DiBAC-expressing *B. subtilis* cells (in green) under a 40× objective for t = 3 h (**D**), t = 5 h (**E**), and t = 7 h (**F**). The images showed a gradual increasing fluorescence intensity from t = 3 h to t = 7 h. Scale bar = 5 µM.

Having confirmed DiBAC as a reliable *Vmem* reporter in *B. subtilis*, we assessed the bioelectrical profile of *B. subtilis* cells after 3, 5, and 7 h of growth (exponential phase, Supplementary Figure S1A) by analyzing the membrane depolarization state of the cells using DiBAC as *Vmem* reporter (Figure 2A). To acquire a better understanding of the results, we consider it convenient to define the *specific growth rate* (*r_t_*), that is, the parameter that describes the cell growth rate at time *t* (see Supplementary Information for a complete explanation and calculations of this parameter).

To determine whether a cell is depolarized or not, we first set a depolarization threshold, defined as the average value of DiBAC fluorescence intensity at t = 3 h of growth (see Image Analysis, Material and Methods). Then, we calculated the percentage of depolarized cells above that threshold to compare the different times (Figure 2B). We observed a significant increase in the percentage of depolarized cells when increasing the culture time (coefficient 0.2059, *p <* 0.001) and the proliferative capacity of the population, but which corresponded to a decreasing *r_t_* at the cell level: 0.01087 at t = 3 h, 0.00738 at t = 5 h and 0.00394 at t = 7 h. Specifically, the population of depolarized cells increased from 38.15 ± 4.96% and 41.04 ± 3.00% at t = 3 h and t = 5 h, respectively, to 57.02 ± 5.83% at t = 7 h. GEE analysis showed *p <* 0.05, *Relative Risk (RR)* = 1.074 between 3 and 5 h; *p <* 0.001, *RR* = 1.530 between 3 and 7 h and *p <* 0.001, *RR* = 1.425 between 5 and 7 h (see Supplementary Table S2 for statistical details; for all groups, *N* = 3 biological replicates with three technical replicates; n = 1943; 2855; and 1924 examined bacteria in 3, 5, and 7 h, respectively).

Additionally, to obtain insight into the variability in the bioelectrical profile within the culture, we analyzed the frequency distribution of DiBAC fluorescence intensities per time, indicating the depolarization threshold at 13.702 arbitrary units (a.u., dashed line; Figure 2C) and representing the data normalized to the number of cells whose intensity value was the most frequent at each time (data taken from the analysis of fluorescence microscopy images; Figure 2D–F)). Our results revealed a slight and gradual shift of the bell curve as time progresses, indicating a higher number of bacterial cells with a fluorescence intensity above the depolarization threshold from t = 3 to t = 7 h. Moreover, the depolarized fraction at t = 5 h and t = 7 h (Figure 2C middle, bottom) seemed to have a similar shape, with a greater number of bacteria showing high fluorescence intensity values. Strikingly, we observed the highest fluorescence intensities at t = 5 h (Figure 2C middle), reaching values of 35 a.u.

These results strongly support that bacterial bioelectrical profile—in terms of membrane depolarization degree—changes throughout the growth dynamics of *B. subtilis*, with an increasing probability of being depolarized as the proliferative properties of the whole bacterial population rise.

### 2.3. Vmem Changes in Response to Neurotransmitter Drugs

**Figure 3:**
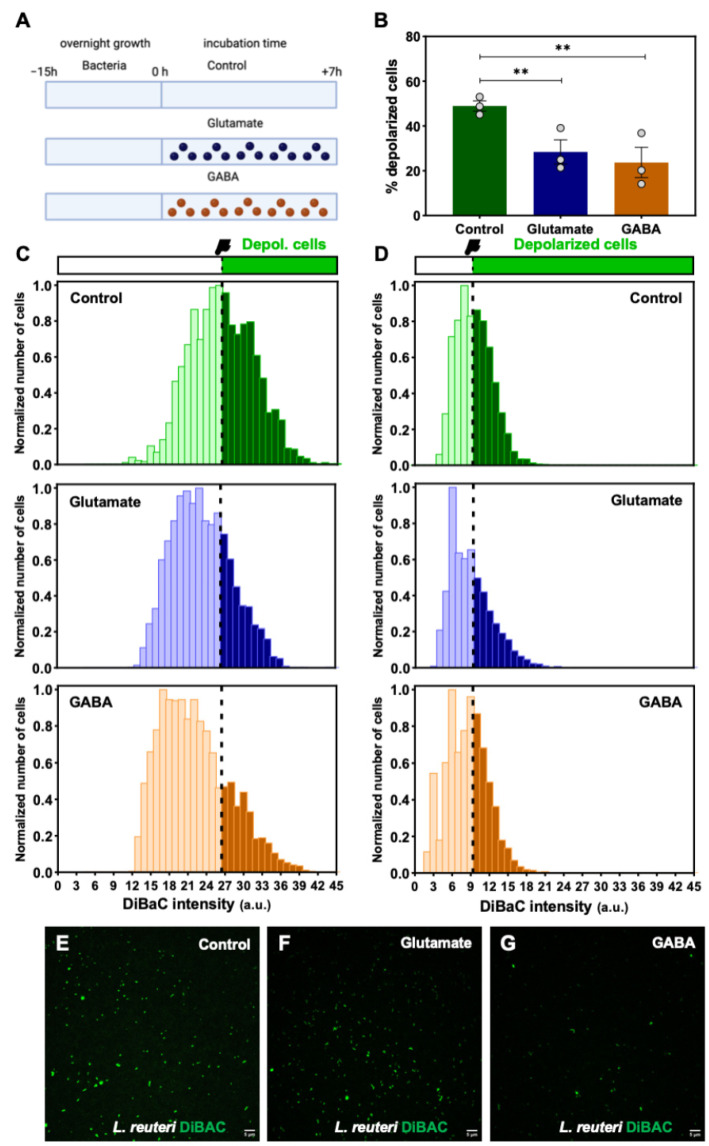
**Effect of the presence of neurotransmitters on the bioelectrical profile of *Bacillus subtilis* (*B. subtilis)* and *Limosilactobacillus reuteri* (*L. reuteri)* cells.** (**A**) Conceptual schematic depicting the experimental procedure of the bacteria-neurotransmitters interaction assay. Pure cultures of *B. subtilis* and *L. reuteri* are grown overnight and inoculated in fresh medium for 7 h of incubation with presence of neurotransmitters. Three treatments are established: Control (with no neurotransmitters in the growth medium), glutamate (bacterial culture with 75 µM glutamate supplemented medium), and GABA (bacterial culture with 0.01 µM GABA supplemented medium). At t = 7 h, bacterial cells of each treatment are sampled and stained with DiBAC. *Vmem* was analyzed by epifluorescence microscopy. Created with BioRender.com. (**B**) Our results showed a significant decrease in the percentage of depolarized cells in neurotransmitters-supplemented cultures compared to the Control. Values from three biological replicates (dots) with three technical replicates are represented per experimental condition. *p* values after applying generalized estimating equations (GEE) statistical method for the percentage of depolarized cells between Control and both glutamate and GABA are indicated as ** *p <* 0.01. (**C**,**D**) Frequency distribution histograms of DiBAC-expressing *B. subtilis* (**C**) and *L. reuteri* (**D**) cells according to their fluorescence intensity for Control (top, green), glutamate-treated (middle, blue), and GABA-treated (bottom, orange) cells. Data are plotted as the total number of cells normalized to the number of those which exhibited the most frequent intensity value in each treatment. Depolarization thresholds set at 26.465 for *B. subtilis* and at 9.350 arbitrary units (a.u) for *L. reuteri* (dashed lines) are calculated as the average DiBAC intensity value of Control cells. (**E**–**G**) Epifluorescence microscopy images showing DiBAC-expressing *L. reuteri* cells (in green) under a 40× objective for Control (**E**), glutamate-treated (**F**), and GABA-treated (**G**) cells. The images showed a higher fluorescence intensity in the Control cells compared to the neurotransmitters-treated cells. Scale bar = 5 µM.

Having demonstrated that *Vmem* and proliferative capacity are linked, and to gain a conceptual understanding of the bacterial response to neurotransmitter effect, we decided to assess the effect of the presence of glutamate and GABA on the percentage of depolarized cells in a *B. subtilis* population. To this end (Figure 3A for a schematic model), the bacteria were then incubated for 7 h in the presence of glutamate and GABA, and the results were compared with a Control group that received no treatment. At the end of the 7-h incubation, the bioelectrical activity of the bacteria in each group was measured using DiBAC as a probe.

We observed a significant effect of neurotransmitters on the bioelectricity of the bacteria (Figure 3B). Our results depicted a decline in the proportion of depolarized bacteria when they were grown in the presence of neurotransmitters (both glutamate and GABA), compared to the Control. In particular, the percentage values ranged from 48.95 ± 2.29% in the Control to 28.46 ± 5.41% and 23.71 ± 6.77% in glutamate and GABA treatments, respectively. The GEE-based statistical study between the Control and glutamate treatment and between the Control and GABA treatment revealed significant differences in both cases (*p <* 0.001), with *RR* = 0.583 and 0.448, respectively (see Supplementary Table S3 for statistical details; for all groups, *N* = 3 biological replicates with three technical replicates; n = 2242, 2257, and 2336 examined bacteria in Control, glutamate and GABA, respectively).

To analyze the depolarization profile of the differently treated cultures, we studied their cell distribution based on their fluorescence intensities (Figure 3C). The bell curve of the Control showed the most widespread distribution of all, with clear differences in number and shape above the depolarization threshold at 26.465 a.u. (dashed line; Figure 3C top) compared to glutamate- and GABA-treated cultures (Figure 3C middle, bottom). In this sense, the bell curve fraction representing the depolarized cell population in glutamate and GABA treatments seemed to be quite distinct in shape, with the GABA-treated bacteria exhibiting greater values of DiBAC fluorescence intensity (Figure 3C bottom).

Having demonstrated the response of *B. subtilis* to the neurotransmitter, we asked whether a gut microbiota strain, *L. reuteri*, with known psychobiotic properties [37], could display specific *Vmem* changes induced by the action of neurotransmitters. As previously validated for *B. subtilis*, first, we checked the DiBAC ability to report *Vmem* changes in controlled KCl-increasing concentrations (with the ionophore Val). Applying GEE, and including as factor “KCl concentration (0, 15, 60, and 300)”, we evaluated the DiBAC-expressing *L. reuteri* population in the four treatments (Supplementary Figure S1B). Our results revealed a significant increase in the percentage of depolarized cells as KCl is increasingly added in the extracellular medium (coefficient 0.002, *p <* 0.001). The percentage of depolarization increased from 22.30 ± 3.50% in the Control group to 30.93 ± 8.20% in Val + KCl 15, to 35.02 ± 6.37% in Val + KCl 60, and to 39.98 ± 6.44% in Val + KCl 300. These results confirm DiBAC as a reliable reporter of depolarization for *L. reuteri*. GEE comparisons between groups showed *p <* 0.001 in all cases (Supplementary Table S4 for statistical details; for all groups, *N* = 3 biological replicates with at least three technical replicates; n = 2556, 2759, 2111, and 1723 examined bacteria in KCl at 0, 15, 60, and 300 mM, respectively).

Once we confirmed the suitability of DiBAC to give us a reliable fluorescence response to *Vmem* changes, we analyzed the effect of the presence of neurotransmitters on *L. reuteri* cells as well (Figure 3D). The results are represented as the number of normalized cells grouped according to their DiBAC intensity, establishing the depolarization threshold (dashed line at 9.35 a.u.) as in the case of *B. subtilis* analysis. We observed different depolarization reduction patterns between glutamate and GABA treatments, the glutamate-treated bacteria being those that exhibited greater values of DiBAC fluorescence intensity. The average percentages comparison between all treatments (Supplementary Figure S1C) revealed a significant decrease in depolarized cell proportion when both glutamate and GABA were present in the medium for *L. reuteri* (a trend like that observed in *B. subtilis* cultures; Figure 3B). In particular, the percentage of depolarized cells declined from 39.89 ± 1.01% for the Control to 27.56 ± 3.64% and 24.98 ± 4.78% for glutamate- and GABA-treated cultures, respectively. The GEE-based statistical study between the Control and both glutamate and GABA treatments revealed significant differences in both cases (*p <* 0.001), with *RR* = 0.724 and 0.722, respectively (see Supplementary Table S5 for statistical details; for all groups, *N* = 3 biological replicates with three technical replicates; n = 6229, 5428, and 5052 examined bacteria in Control-, glutamate-, and GABA-treated groups). All these general trends in membrane depolarization patterns were quite evident in epifluorescence microscopy images (Figure 3E–G).

Considering all these data, we claim that the presence of neural stimuli such as neurotransmitters causes changes in bacterial bioelectrical profile in *B. subtilis* and *L. reuteri* cells by reducing their membrane depolarization.

## 3. Discussion

The existence of bi–directional communication between the gut and the brain (the gut–brain axis) has long been recognized [38,39]. Since the established pathways of gut–brain communication encompass mainly the neural pathway [40], traditionally, the interest of researchers was focused on the uni-directional influence of the gut and its microbiota on the nervous system (especially the enteric nervous system; ENS), and, from a clinical perspective, on how various diseases that affect the alimentary tract (e.g., IBS, inflammatory gut disorders, anorexia nervosa, and obesity) may dysregulate the gut–brain axis [41]. More recently, the existence of an intense crosstalk between the microbes in the gut and the ENS has emerged [42,43]. The microbiota can modulate neonatal brain development [44], host behavior [45], and cognitive properties [46], leading to our rethinking the gut microbiota—brain axis in favor of a comprehensive bacteria–brain inter-kingdom communication [26,47,48]. From a clinical perspective, growing evidence has been accumulated on the potential role of alterations of gut microbiota in the pathogenesis and/or symptomatology of major psychiatric disorders, demanding more research for the better understanding of the mechanistic links along the gut microbiota–brain axis.

From a therapeutic perspective, various approaches for treating the microbiota have been explored, including targeting a broad spectrum of molecular and cellular elements [49], such as manipulating the activity of Toll-like receptors (TLR), since the microbiota produces several TLR ligands related to the development of inflammatory processes [50] or the use of probiotics for regulating tryptophan and serotonin metabolism [51]. Yet, the potential impact of lifestyle interventions (such as diet and exercise) on physical and mental health through gut–microbiota-mediated actions have been recognized [52]. It has been shown that exercise has an impact on *Firmicutes* and *Actinobacteria* bacterial phyla [53,54] (which include the *Lactobacillus* and *Bifidobacterium* genera) and induces changes in the microbial production of SCFAs, specifically butyrate [55], which may regulate anxiety levels [56]. However, when considering the high variability of psychobiotic properties, without first increasing our understanding of brain–bacteria communication, we depend on trial and error [57].

All types of cells, and not only electrogenic cells (such as neurons and cardiomyocytes), have the ability to establish a difference in electrical charges across its cellular membrane. This membrane potential (*Vmem*), alongside its role as a source of energy, is increasingly being recognized as a mechanism by which cells can actively regulate a wide range of physiological events. Nowadays, we know that bacteria utilize their *Vmem* as a a means of signaling and processing information, in a similar way to neurons and glial cells [3,16,17,26,58] (for an excellent review on membrane potential dynamics in bacteria, see [13]). The use of voltage sensitive dyes to reveal the relevant properties of cell physiology and inter-cellular communication in different animal models is growing in popularity [34,59,60,61,62,63,64]. *Vmem* Nerstian dyes (Figure 1A), such as DiSC3(5), Thioflavin T (ThT), and DiBAC, have been employed to evaluate *Vmem* dynamics in both Gram-negative and Gram-positive bacteria [65]. Cells depolarize or hyperpolarize in a dynamic basis in response to molecular, cellular, and population events, such as motility [66], antibiotic resistance [14,15], biofilm communication [3,16], cell division [67], and environmental perception [68]. Hence, developing and testing experimental methods to measure bacterial *Vmem* is pivotal to increasing our knowledge and to gaining new insights into the potential of manipulating the bioelectrical properties of bacteria for microbiota-targeted interventions in psychiatric diseases (*electroceutical* approaches as coined in [12]).

The results of this study show that the proliferative capacity of a *B. subtilis* population is linked to an increase in depolarization (Figure 2). Strikingly, as the specific growth rate (*rt*; see Supplementary Information for details) is decreasing (from 3 h to 7 h), which means that individual cells are less energized but the population is growing, there is a clear rise in the percentage of depolarized cells. Other than being a free energy source [13], the dynamic changes in the endogenous bioelectrical properties might carry instructive cues, as has been previously demonstrated in other proliferative processes, such as development and cancer progression (tumors have depolarized bioelectric signatures [64]). Interestingly, an external electrical stimulus induces opposite changes in bacteria *Vmem* depending on the state of the cells [69]. Yet, we characterize the dynamics of the *Vmem* in *B. subtilis* during the different states of the curve growth, stating a clear increase in depolarization. Whether this fact is mediated by the potassium ion channel YugO (as it is involved in depolarization induced by external electrical stimulation in *Escherichia coli* [69]) remains unknown, and future experiments will shed light on it. This proof-of-principle linking *Vmem* properties and proliferative capacity in bacteria opens different important questions; for example: Do bioelectrical gradients within the cell population carry information about patterning and/or growth? Can we alter the proliferative abilities of the culture by altering some channels? Do the cells of the culture respond equally? The methodology described in this paper will allow us to address these questions and will eventually increase our understanding of bacterial electrical signaling.

Whereas the possibility that bacteria can exert effects on neurons through neurotransmitter production and/or modulation is well recognized [70,71], little is known about the effects that neurotransmitters exert on bacteria. Here, we focused on the effects of glutamate and GABA—the two main neurotransmitters of the CNS and widely present in the gut microbiota–brain axis—on the bioelectric signature of two different bacterial strains: *B. subtilis* and *L. reuteri*. *B. subtilis* was chosen as it has been the most recognized and most widely used strain in the study bioelectrical signaling in bacteria since Prindle et al. described its capacity for cell-to-cell bioelectrical communication in 2015, being the first bacterium in which it was discovered [3,65,69]. Then, to acquire a broader view of the effect of neurotransmitters in bacteria, we included *L. reuteri* in the study, a gut microbiota species and a well-known probiotic [37], which is metabolically and physiologically very different from *B. subtilis*.

The results obtained via our methodological approach for detecting *Vmem* changes in bacteria showed a depolarization effect of both glutamate and GABA in bacteria (Figure 3), despite having antagonistic effects in the nervous system of mammals. Neurotransmitters are most often investigated for their role in carrying information throughout the nervous system; however, importantly, they also act as chemical messengers in many signaling pathways of non-neuronal origin as they precede the appearance of nervous systems on both developmental and evolutionary time scales. Neurotransmitters can act as morphogens [72], as regulators for migration of tumor cells [73] and immune mediators [74], or as mediators in embryogenesis and regeneration [75], among other roles involving non-neural structures. The aligned response in bacteria induced by both glutamate and GABA does not necessarily imply that the bioelectrical signature of bacteria is insensitive to which neurotransmitter-related pathway is inhibited or activated; in fact, similar effects caused by antagonistic drugs are not uncommon outside of the nervous system of mammals. For example, a number of molecular pathways do not show opposite phenotypes from agonists and antagonists in animal models such as *Xenopus* (i.e., the same craniofacial and patterning defects are obtained from agonist and antagonist drugs [75]).

On average, the changes in the bioelectrical signature of bacteria are quantitively similar when comparing the effects of the two neurotransmitters. However, an in-depth analysis of cell distribution based on fluorescence intensity (Figure 3C,D) showed that the bell curve fraction representing the depolarized cell population is different, pointing to GABA as the treatment that induces greater depolarization values in bacteria. Bacterial cells could be influenced by optimal ranges for specific signals, outside of which cell behavior is disrupted in similar ways. Bacteria possess a broad range of ion channels [76], including mechanosensitive channels [77], K^+^ channels, Na^+^ channels, Cl^-^ channels, cyclic nucleotide gated channels-gated channels, and glutamate receptor channels [78]. Glutamate is the gating molecule for the YugO channel, which mediates electrical signaling within a *B. subtilis* biofilm [3]. Moreover, glutamate receptors are implied in the propagation of electrical activity in plants [4], and they are related to molecular bioelectricity in *Xenopus* regeneration [75]. Likewise, GABA production by microbiota bacteria is well known [79]. GABA might act as a molecule of communication between bacteria [80], and influence mobility, growth kinetic, and biofilm formation activity [81]. This evidence and similar *Vmem* responses to neurotransmitters between two different bacterial species (*B. subtilis* and *L. reuteri*) provide empirical support for the view that bacteria activity can be influenced by neurotransmitters (of neuronal origin and/or produced by bacteria themselves), implying changes in bioelectric cues detected for other organisms.

Whether the neurotransmitter-induced decrease in depolarization is activating or inhibiting bacteria remains unclear, and future experiments are needed in order to explore this issue. Depolarized bacteria seem to be more resistant to antibiotic effects [82]. Conversely, external electrical stimulation induces the opposite effects on activated cells (hyperpolarization) to those induced in inhibited cells (depolarization [69]), and hyperpolarized energized cells have been described [65]. Despite the obvious limitations of in vitro assays, we foresee that combining neurochemical and electrical stimulation [83] in bacteria, coupled with *Vmem* readings, will allow us to monitor and decode the bidirectional mechanisms by which neural signals can act on bacterial cells (in emerging technologies such as Organ-on-a-Chip (OoC), thus addressing whether and how the dynamics of *Vmem* under growth conditions and neurotransmitter stimuli lead to cellular downstream cascades and changes in gene expression. To extend our results, further studies testing our hypotheses in other bacterial species, including Gram-negative strains, are needed. In addition, we have focused on bacteria since they are the major component of the gut microbiome and the most widely related to the gut microbiota–brain axis. To gain insight into the potential role of other relevant components of the microbiome (such as mycobiome, virome, and archaeome), a key methodological issue which might be overcome using the techniques described in this paper is the detection of the bioelectrical profile of these micro-organisms.

In conclusion, we have described and tested experimentally an innovative methodology with which to investigate the dynamics of membrane potentials in bacteria in response to neural stimuli. Our goal was to establish a proof-of-principle assay, a set of multi-factorial analysis metrics, and a baseline set of results to facilitate the future screening and evaluation of novel interventions targeting the bioelectrical properties of gut microbiota that could influence bacteria–neural communication. Our results demonstrate that the dynamics of *Vmem* in bacteria can be a reliable read-out to the actions of neural-type external stimuli. This makes it possible to further elucidate the mechanisms underlying electrical signaling in bacteria, opening exciting perspectives in the study of the gut microbiota–brain axis and, eventually, in the design of effective microbiota-targeted interventions for psychiatric diseases.

## 4. Material and Methods

### 4.1. Bacterial Strains and Growth Conditions

The strains used in this study were *Bacillus subtilis* Marburg ATCC 6051 (*B. subtilis*) and *Limosilactobacillus reuteri* F275 ATCC 23272 (*L. reuteri*). Both strains were stored at −80 °C in cryopreservation medium until use. The stock was then used to prepare cultures in liquid media: Trypticasein Soy Broth (TSB, Condalab; Madrid, Spain) for *B. subtilis* and Man Rogosa Sharpe (MRS, Condalab; Madrid, Spain) for *L. reuteri*. In all experiments, *B. subtilis* was grown at 37 °C with O_2_. *L. reuteri* was grown at 37 °C in a microaerophilic environment. Microaerophilic conditions were achieved by setting a 1:5 air:culture ratio in the flasks, without agitation.

### 4.2. Calibration of Depolarizing Medium by Increasing KCl Concentrations

To validate DiBAC as a *Vmem* reporter, bacteria were placed in depolarizing conditions. We created the depolarizing conditions considering the Nernst Equation for $K^{+}$ equilibrium potential (*V_Eq__._*; see Supplementary Information for a complete explanation of equation details). Increasing concentrations of KCl were added in the extracellular medium $\left({\left[K^{+}\right]}_{out}\right)$ until reaching *Vmem* ∼0 mV.

(1) $$V_{Eq.}=\frac{RT}{zF}ln\left(\frac{{\left[K^{+}\right]}_{out}}{{\left[K^{+}\right]}_{in}}\right)$$

To do this, we added KCl until reaching $\left({\left[K^{+}\right]}_{out}\right)$ = 0, 15, 60, and 300 mM, which corresponds to *V_Eq._* = −110, −75, −40, and 0 mV, respectively. In resting conditions (with KCl 0 mM), $\left({\left[K^{+}\right]}_{out}\right)$ is ∼3mM (in 1x PBS). A total of 300 mM of KCl was used as an approximation for the cellular $K^{+}$ concentration $\left({\left[K^{+}\right]}_{in}\right)$ of *B. subtilis* cells [84]. To increase the cell membrane permeability to $K^{+}$ ions, 5 µM valinomycin (Val; Fisher Scientific ref. V1644; Madrid, Spain) was used as an ionophore or $K^{+}$ carrier (Figure 1A).

### 4.3. DiBAC4(3) Validation as a Reporter of Vmem Changes (Depolarization)

*B. subtilis* was grown overnight at 37 °C with O_2_ in TSB and sub-cultured in fresh medium for an additional 12–16 h (h) until the optical density at 600 nm (OD_600_) reached ~0.8, which corresponds to the late exponential phase of growth. At this point, cells were centrifuged (2000× *g*, 10 min, room temperature—RT) and resuspended in phosphate-buffered saline (PBS, 1x), diluting the sample to an OD_600_ ~0.3. These cells were placed in a 48-well plate with 5 µM Val and 15, 60, or 300 mM of KCl. Cells incubated without KCl were analyzed as a Control as well. Before epifluorescence microscopy analysis, cells of each sample were stained with 100 µM of bis-(1,3-dibutylbarbituric acid) trimethine oxonol) or DiBAC4(3) (DiBAC; Fisher Scientific ref. B438; Madrid, Spain) and incubated for 15 min at RT in the dark. Dye concentration (100 µM) and staining conditions were selected after an optimization process wherein different DiBAC concentrations and incubation times and temperatures were evaluated.

### 4.4. Bioelectrical Analysis of B. subtilis at Growth Dynamics

*B. subtilis* was grown overnight with O_2_ at 37 °C in TSB and sub-cultured in fresh medium for 12–16 h. The OD_600_ of the culture was then adjusted to 0.01 in fresh TSB and incubated at 37 °C. After 3, 5, and 7 h of incubation, OD of 1 mL aliquots was measured, and the bioelectrical analysis was made (Figure 2A). Cells were centrifuged (2000× *g*, 10 min, RT), resuspended in 1x PBS, diluted to an OD_600_ ~0.3, and placed in a 48-well plate with 100 µM DiBAC. After 15 min at RT in the dark, the epifluorescence microscopy analysis was made.

### 4.5. Effect of Neurotransmitters on B. subtilis and L. reuteri Cells

The preparation of both cultures was performed as described above. Once the mid-late exponential phase of growth was reached, the OD_600_ was measured and adjusted to 0.01 in fresh TSB and MRS media (for *B. subtilis* and *L. reuteri*, respectively). For glutamate and GABA assays, cell suspensions were supplemented with 75 µM of glutamate (Tocris-Biotechne, Bio-Techne R&D Systems, S.LU, ref. 0218; Madrid, Spain and 0.01 µM of GABA (Tocris-Biotechne, Bio-Techne R&D Systems, S.LU, ref.0344; Madrid, Spain), respectively, and incubated at 37 °C under same conditions (Figure 3A). After 7 h of drug incubation, cells were centrifuged (2000× *g*, 10 min, RT), resuspended in 1x PBS diluting the sample to an OD ~0.3 and placed in a 48-well plate with 100 µM (for *B. subtilis*) and 200 µM (for *L. reuteri*) DiBAC. When using a voltage-sensitive dye on new bacterial species, the optimal dye concentration and incubation time should first be determined, as extensively showed in [65], as different species do not respond equally to same dye conditions. After 15 min at RT in the dark, the epifluorescence microscopy analysis was made. Untreated glutamate and GABA cells were established as a Control group. Drug concentrations were determined using ranges supported by the supplier and through dose screening and were applied at levels that did not result in observable toxic effects.

### 4.6. DiBAC Imaging

For both DiBAC validation and bioelectrical analysis of growth dynamics and neurotransmitter effect assays, 5 µL of the DIBAC-stained cells solution was placed onto a microscope slide and covered with a 19 mm diameter microscopy coverslip. A Leica DMi8 (Leica microsystems; Milano, Italy) inverted microscope was used. A FITC LP filter was used for an excitation wavelength of 450/490 nm with an exposure time of 30 ms. Paired images of at least five random fields were taken in each sample, both in brightfield (BF) and under FITC filters.

### 4.7. Image Analysis

A custom-written FIJI macro (ImageJ; National Institutes of Health, Bethesda, MD, USA) was used to identify bacterial cells on phase-contrast images to create a mask for application on the FITC channel (Figure 1B). Non-cellular particulates and background noise were eliminated through size filtering and fluorescence intensity for each individual cell measured. Using the DiBAC average fluorescence intensity from Control bacteria (t = 3 h for physiological assays and never exposed to drugs for neurotransmitter assays), we set the depolarization threshold at the mean value. Per each replicate and condition, we calculated the percentage of cells above the depolarization threshold from DiBAC images related to BF images. Per each condition, we used three independent biological replicates with three technical replicates and at least five images per sample. Histograms were generated by combining data from different replicates of the same condition.

### 4.8. Statistical Analysis

To study the bacterial response (i.e., depolarization), generalized estimating equations (GEE) were used. To do this, the experimental variations in the proportion of depolarized cells among the different experimental groups (treatments or times) were evaluated. To compare how much the probability of depolarization changes (increases or decreases) between treatments, we use *logit* as the link function, thus creating multilevel logistic regression models (MLLR). In these models, the biological replicate was chosen as the grouping variable (panel variable) to consider the possible dependency of the data. The statistical analyses used, *p*-values, and the number of replicate measurements (*N*) are stated in the Results section and each figure legend. For all cases, a minimum of three biological replicates consisting of three technical replicates were used. Unless otherwise indicated, data are represented as mean ± standard error of the mean (SEM). The significance level was set to 0.05 in all cases. Statistical analysis and graphs were performed using STATA 2017 (*Stata Statistical Software: Release 15*., College Station, TX, USA) and GraphPad Prism v. 8.0.2.(GraphPad Software, Inc., Boston, MA, USA).

## Data Availability

Further information and requests for reagents may be directed to, and will be fulfilled by, the Lead Contact Celia Herrera-Rincon (ceherrer@ucm.es).

## Supplementary Materials

The following supporting information, including one figure and five tables can be downloaded at: https://www.mdpi.com/article/10.3390/ijms241713394/s1.

## References

[1] Hotary K.B., Robinson K.R. (1992). Evidence of a role for endogenous electrical fields in chick embryo development. Development.

[2] Levin M. (2012). Molecular bioelectricity in developmental biology: New tools and recent discoveries: Control of cell behavior and pattern formation by transmembrane potential gradients. BioEssays.

[3] Prindle A., Liu J., Asally M., Ly S., Garcia-Ojalvo J., Süel G.M. (2015). Ion channels enable electrical communication in bacterial communities. Nature.

[4] Hedrich R., Salvador-Recatalà V., Dreyer I. (2016). Electrical Wiring and Long-Distance Plant Communication. Trends Plant Sci..

[5] Adamatzky A. (2022). Language of fungi derived from their electrical spiking activity. R. Soc. Open Sci..

[6] Levin M. (2014). Molecular bioelectricity: How endogenous voltage potentials control cell behavior and instruct pattern regulation in vivo. Mol. Biol. Cell.

[7] Zhao M., Song B., Pu J., Wada T., Reid B., Tai G., Wang F., Guo A., Walczysko P., Gu Y. (2006). Electrical signals control wound healing through phosphatidylinositol-3-OH kinase-γ and PTEN. Nature.

[8] Fukumoto T., Kema I.P., Levin M. (2005). Serotonin signaling is a very early step in patterning of the left-right axis in chick and frog embryos. Curr. Biol..

[9] Jenkins L.S., Duerstock B.S., Borgens R.B. (1996). Reduction of the current of injury leaving the amputation inhibits limb regeneration in the red spotted newt. Dev. Biol..

[10] Yang M., Brackenbury W.J. (2013). Membrane potential and cancer progression. Front. Physiol..

[11] Cervera J., Manzanares J.A., Levin M., Mafe S. (2023). Transplantation of fragments from different planaria: A bioelectrical model for head regeneration. J. Theor. Biol..

[12] Pio-Lopez L., Levin M. (2023). Morphoceuticals: Perspectives for discovery of drugs targeting anatomical control mechanisms in regenerative medicine, cancer and aging. Drug Discov. Today.

[13] Benarroch J.M., Asally M. (2020). The Microbiologist’s Guide to Membrane Potential Dynamics. Trends Microbiol..

[14] Liu J., Prindle A., Humphries J., Gabalda-Sagarra M., Asally M., Lee D.Y.D., Ly S., Garcia-Ojalvo J., Süel G.M. (2015). Metabolic co-dependence gives rise to collective oscillations within biofilms. Nature.

[15] Liu J., Martinez-Corral R., Prindle A., Lee D.-Y.D., Larkin J., Gabalda-Sagarra M., Garcia-Ojalvo J., Süel G.M. (2017). Coupling between distant biofilms and emergence of nutrient time-sharing. Science.

[16] Humphries J., Xiong L., Liu J., Prindle A., Yuan F., Arjes H.A., Tsimring L., Süel G.M. (2017). Species-Independent Attraction to Biofilms through Electrical Signaling. Cell.

[17] Yang C.Y., Bialecka-Fornal M., Weatherwax C., Larkin J.W., Prindle A., Liu J., Garcia-Ojalvo J., Süel G.M. (2020). Encoding Membrane-Potential-Based Memory within a Microbial Community. Cell Syst..

[18] Bercik P., Collins S.M., Verdu E.F. (2012). Microbes and the gut-brain axis. Neurogastroenterol. Motil..

[19] Mayer E.A., Tillisch K., Gupta A. (2015). Gut/brain axis and the microbiota. J. Clin. Investig..

[20] Cryan J.F., O’Riordan K.J., Cowan C.S., Sandhu K.V., Bastiaanssen T.F., Boehme M., Codagnone M.G., Cussotto S., Fulling C., Golubeva A.V. (2019). The Microbiota-Gut-Brain Axis. Physiol. Rev..

[21] Lee J.Y., Tsolis R.M., Bäumler A.J. (2022). The microbiome and gut homeostasis. Science.

[22] Liu F., Li J., Wu F., Zheng H., Peng Q., Zhou H. (2019). Altered composition and function of intestinal microbiota in autism spectrum disorders: A systematic review. Transl. Psychiatry.

[23] Wu S.C., Cao Z.S., Chang K.M., Juang J.L. (2017). Intestinal microbial dysbiosis aggravates the progression of Alzheimer’s disease in Drosophila. Nat. Commun..

[24] Bedarf J.R., Hildebrand F., Coelho L.P., Sunagawa S., Bahram M., Goeser F., Bork P., Wüllner U. (2017). Functional implications of microbial and viral gut metagenome changes in early stage L-DOPA-naïve Parkinson’s disease patients. Genome Med..

[25] Dinan T.G., Cryan J.F. (2017). The Microbiome-Gut-Brain Axis in Health and Disease. Gastroenterol. Clin..

[26] Murciano-Brea J., Garcia-Montes M., Geuna S., Herrera-Rincon C. (2021). Gut microbiota and neuroplasticity. Cells.

[27] Petroff O.A.C. (2002). GABA and glutamate in the human brain. Neuroscientist.

[28] Conn P.J. (2003). Physiological Roles and Therapeutic Potential of Metabotropic glutamate Receptors. Ann. N. Y. Acad. Sci..

[29] Pessione E. (2012). Lactic acid bacteria contribution to gut microbiota complexity: Lights and shadows. Front. Cell. Infect. Microbiol..

[30] Tsai M.F., McCarthy P., Miller C. (2013). Substrate selectivity in glutamate-dependent acid resistance in enteric bacteria. Proc. Natl. Acad. Sci. USA.

[31] Mazzoli R., Pessione E. (2016). The Neuro-endocrinological Role of Microbial glutamate and GABA Signaling. Front. Microbiol..

[32] Baj A., Moro E., Bistoletti M., Orlandi V., Crema F., Giaroni C. (2019). glutamatergic signaling along the microbiota-gut-brain axis. Int. J. Mol. Sci..

[33] Cohen A.E., Venkatachalam V. (2014). Bringing bioelectricity to light. Annu. Rev. Biophys..

[34] Adams D.S., Levin M. (2012). Measuring resting membrane potential using the fluorescent voltage reporters DiBAC4 and CC2-DMPE. Cold Spring Harb. Protoc..

[35] Sträuber H., Müller S. (2010). Viability states of bacteria-Specific mechanisms of selected probes. Cytom. Part A.

[36] Maher M.P., Wu N.T., Ao H. (2007). pH-Insensitive FRET Voltage Dyes. J. Biomol. Screen..

[37] Haas-Neill S., Iwashita E., Dvorkin-Gheva A., Forsythe P. (2022). Effects of Two Distinct Psychoactive Microbes, *Lacticaseibacillus rhamnosus* JB-1 and *Limosilactobacillus reuteri* 6475, on Circulating and Hippocampal mRNA in Male Mice. Int. J. Mol. Sci..

[38] Foster J.A., McVey Neufeld K.A. (2013). Gut-brain axis: How the microbiome influences anxiety and depression. Trends Neurosci..

[39] Kundu P., Blacher E., Elinav E., Pettersson S. (2017). Our Gut Microbiome: The Evolving Inner Self. Cell.

[40] Naveed M., Zhou Q.-G., Xu C., Taleb A., Meng F., Ahmed B., Zhang Y., Fukunaga K., Han F. (2021). Gut-brain axis: A matter of concern in neuropsychiatric disorders…!. Prog. Neuropsychopharmacol. Biol. Psychiatry.

[41] Parekh P.J., Balart L.A., Johnson D.A. (2015). The influence of the gut microbiome on obesity, metabolic syndrome and gastrointestinal disease. Clin. Transl. Gastroenterol..

[42] Mayer E.A. (2011). Gut feelings: The emerging biology of gut–brain communication. Nat. Rev. Neurosci..

[43] Wiley N.C., Cryan J.F., Dinan T.G., Ross R.P., Stanton C. (2021). Production of Psychoactive Metabolites by Gut Bacteria. Mod. Trends Psychiatry.

[44] Gars A., Ronczkowski N.M., Chassaing B., Castillo-Ruiz A., Forger N.G. (2021). First encounters: Effects of the microbiota on neonatal brain development. Front. Cell. Neurosci..

[45] Vuong H.E., Yano J.M., Fung T.C., Hsiao E.Y. (2017). The microbiome and host behavior. Annu. Rev. Neurosci..

[46] Chu C., Murdock M.H., Jing D., Won T.H., Chung H., Kressel A.M., Tsaava T., Addorisio M.E., Putzel G.G., Zhou L. (2019). The microbiota regulate neuronal function and fear extinction learning. Nature.

[47] Le Chatelier E., Nielsen T., Qin J., Prifti E., Hildebrand F., Falony G., Almeida M., Arumugam M., Batto J.-M., Kennedy S. (2013). Richness of human gut microbiome correlates with metabolic markers. Nature.

[48] Lyon P., Keijzer F., Arendt D., Levin M. (2021). Reframing cognition: Getting down to biological basics. Philos. Trans. R. Soc. B Biol. Sci..

[49] Spichak S., Bastiaanssen T.F.S., Berding K., Vlckova K., Clarke G., Dinan T.G., Cryan J.F. (2021). Mining microbes for mental health: Determining the role of microbial metabolic pathways in human brain health and disease. Neurosci. Biobehav. Rev..

[50] Caputi V., Giron M.C. (2018). Microbiome-gut-brain axis and toll-like receptors in Parkinson’s disease. Int. J. Mol. Sci..

[51] O’Mahony S.M., Clarke G., Borre Y.E., Dinan T.G., Cryan J.F. (2015). Serotonin, tryptophan metabolism and the brain-gut-microbiome axis. Behav. Brain Res..

[52] Gubert C., Kong G., Renoir T., Hannan A.J. (2020). Exercise, diet and stress as modulators of gut microbiota: Implications for neurodegenerative diseases. Neurobiol. Dis..

[53] Dalton A., Mermier C., Zuhl M. (2019). Exercise influence on the microbiome-gut-brain axis. Gut Microbes.

[54] Li Y., Zafar S., Salih Ibrahim R.M., Chi H.L., Xiao T., Xia W.J., Li H.B., Kang Y.M. (2021). Exercise and food supplement of vitamin C ameliorate hypertension through improvement of gut microflora in the spontaneously hypertensive rats. Life Sci..

[55] Allen J.M., Mailing L.J., Niemiro G.M., Moore R., Cook M.D., White B.A., Holscher H.D., Woods J.A. (2018). Exercise alters gut microbiota composition and function in lean and obese humans. Med. Sci. Sports Exerc..

[56] Kang S.S., Jeraldo P.R., Kurti A., Miller M.E., Cook M.D., Whitlock K., Goldenfeld N., Woods J.A., White B.A., Chia N. (2014). Diet and exercise orthogonally alter the gut microbiome and reveal independent associations with anxiety and cognition. Mol. Neurodegener..

[57] Long-Smith C., O’Riordan K.J., Clarke G., Stanton C., Dinan T.G., Cryan J.F. (2020). Microbiota-gut-brain axis: New therapeutic opportunities. Annu. Rev. Pharmacol. Toxicol..

[58] Martinez-Corral R., Liu J., Prindle A., Süel G.M., Garcia-Ojalvo J. (2019). Metabolic basis of brain-like electrical signalling in bacterial communities. Philos. Trans. R. Soc. B Biol. Sci..

[59] Chernet B., Levin M. (2013). Endogenous Voltage Potentials and the Microenvironment: Bioelectric Signals That Reveal, Induce and Normalize Cancer. J. Clin. Exp. Oncol..

[60] Tseng A., Levin M. (2013). Cracking the bioelectric code: Probing endogenous ionic controls of pattern formation. Commun. Integr. Biol..

[61] Levin M., Mercola M. (1998). Gap junctions are involved in the early generation of left-right asymmetry. Dev. Biol..

[62] Adams D.S., Levin M. (2013). Endogenous voltage gradients as mediators of cell-cell communication: Strategies for investigating bioelectrical signals during pattern formation. Cell Tissue Res..

[63] McLaughlin K.A., Levin M. (2018). Bioelectric signaling in regeneration: Mechanisms of ionic controls of growth and form. Dev. Biol..

[64] Levin M. (2021). Bioelectric signaling: Reprogrammable circuits underlying embryogenesis, regeneration, and cancer. Cell.

[65] Te Winkel J.D., Gray D.A., Seistrup K.H., Hamoen L.W., Strahl H. (2016). Analysis of antimicrobial-triggered membrane depolarization using voltage sensitive dyes. Front. Cell Dev. Biol..

[66] Miller J.B., Koshland D.E. (1977). Sensory electrophysiology of bacteria: Relationship of the membrane potential to motility and chemotaxis in *Bacillus subtilis*. Proc. Natl. Acad. Sci. USA.

[67] Strahl H., Hamoen L.W. (2010). Membrane potential is important for bacterial cell division. Proc. Natl. Acad. Sci. USA.

[68] Kikuchi K., Galera-Laporta L., Weatherwax C., Lam J.Y., Chae Moon E., Theodorakis E.A., Garcia-Ojalvo J., Süel G.M. (2022). Electrochemical potential enables dormant spores to integrate environmental signals. Science.

[69] Stratford J.P., Edwards C.L.A., Ghanshyam M.J., Malyshev D., Delise M.A., Hayashi Y., Asally M. (2019). Electrically induced bacterial membrane-potential dynamics correspond to cellular proliferation capacity. Proc. Natl. Acad. Sci. USA.

[70] Strandwitz P. (2018). Neurotransmitter modulation by the gut microbiota. Brain Res..

[71] Dicks L.M.T. (2022). Gut Bacteria and Neurotransmitters. Microorganisms.

[72] Lauder J.M. (1988). Neurotransmitters as morphogens. Prog. Brain Res..

[73] Entschladen F., Lang K., Drell T.L., Joseph J., Zaenker K.S. (2022). Neurotransmitters are regulators for the migration of tumor cells and leukocytes. Cancer Immunol. Immunother..

[74] Herrera-Rincon C., Paré J.F., Martyniuk C.J., Jannetty S.K., Harrison C., Fischer A., Dinis A., Keshari V., Novak R., Levin M. (2020). An in vivo brain–bacteria interface: The developing brain as a key regulator of innate immunity. NPJ Regen. Med..

[75] Sullivan K.G., Levin M. (2016). Neurotransmitter signaling pathways required for normal development in Xenopus laevis embryos: A pharmacological survey screen. J. Anat..

[76] Martinac B., Saimi Y., Kung C. (2008). Ion Channels in Microbes. Physiol. Rev..

[77] Bruni G.N., Weekley R.A., Dodd B.J.T., Kralj J.M. (2017). Voltage-gated calcium flux mediates *Escherichia coli* mechanosensation. Proc. Natl. Acad. Sci. USA.

[78] Chen C.G., Cui C., Mayer M.L., Gouaux E. (1999). Functional characterization of a potassium-selective prokaryotic glutamate receptor. Nature.

[79] Strandwitz P., Kim K.H., Terekhova D., Liu J.K., Sharma A., Levering J., McDonald D., Dietrich D., Ramadhar T.R., Lekbua A. (2019). GABA-modulating bacteria of the human gut microbiota. Nat. Microbiol..

[80] Sarasa S.B., Mahendran R., Muthusamy G., Thankappan B., Selta D.R.F., Angayarkanni J. (2020). A Brief Review on the Non-protein Amino Acid, Gamma-amino Butyric Acid (GABA): Its Production and Role in Microbes. Curr. Microbiol..

[81] Dagorn A., Chapalain A., Mijouin L., Hillion M., Duclairoir-Poc C., Chevalier S., Taupin L., Orange N., Feuilloley M.G.J. (2013). Effect of GABA, a bacterial metabolite, on *Pseudomonas fluorescens* surface properties and cytotoxicity. Int. J. Mol. Sci..

[82] Lee D.Y.D., Galera-Laporta L., Bialecka-Fornal M., Moon E.C., Shen Z., Briggs S.P., Garcia-Ojalvo J., Süel G.M. (2019). Magnesium Flux Modulates Ribosomes to Increase Bacterial Survival. Cell.

[83] Comerci C.J., Gillman A.L., Galera-Laporta L., Gutierrez E., Groisman A., Larkin J.W., Garcia-Ojalvo J., Süel G.M. (2022). Localized electrical stimulation triggers cell-type-specific proliferation in biofilms. Cell Syst..

[84] Whatmore A.M., Reeds R.H. (1990). Determination of turgor pressure in *Bacillus subtilis*: A possible role for K^+^ in turgor regulation. J. Gen. Microbiol..

