# Supplementary material for: Bioelectrical State of Bacteria Is Linked to Growth Dynamics and Response to Neurotransmitters: Perspectives for the Investigation of the Microbiota–Brain Axis
Source: Int J Mol Sci. 2023 Aug 29;24(17):13394. doi: 10.3390/ijms241713394 (PMC10488255; doi:10.3390/ijms241713394)
Supplement: Supplementary file 1 [file ijms-24-13394-s001.zip › ijms-2554693-supplementary.pdf]

Supplementary Data

SUPPLEMENTARY FIGURES

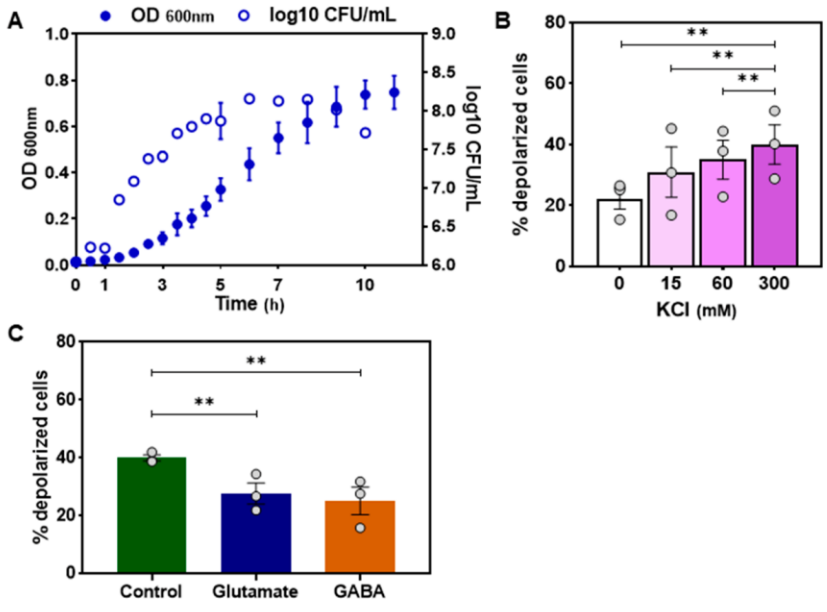

Supplementary Figure 1

Suppl. Fig. 1(*A*) Growth dynamics of *Bacillus subtilis* (*B. subtilis*) ~~(~~growing in Trypticasein Soy Broth (TSB) medium, aerobic conditions, ~~)~~ in terms of optical density at 600 nm (OD600; ~~OD600 (~~blue dots) and log CFU/mL (white dots). Mean values from three biological replicates are represented. Error bars correspond to the standard error of the mean (SEM). **(*B*)** DiBAC validation assay for *Limosilactobacillus reuteri* (*L. reuteri*) showed significant increases in the percentage of depolarized cells (DiBAC) as ~~the~~ potassium chloride (KCl) increases in the extracellular medium. Values from three biological replicates (dots) with, at least, three technical replicates, for each condition are plotted per experimental condition. *P* values after applying generalized estimating equations (GEE) for percentage of depolarized cells among all groups are indicated as ** *P* <0.01. (***C***) Effect of the presence of glutamate (75 µM) and GABA (0.01 µM) on the percentage of depolarized cells of *L. reuteri*, compared to the Control group (with no ~~neurotrasmitters~~neurotransmitters). The average percentages are shown. Error bars correspond to the ~~standard error of the mean (SEM)~~SEM. Experiments were performed in triplicate. DiBAC validation assay for *L. reuteri* showed significant increases in the percentage of depolarized cells (DiBAC) as the KCl increases in the extracellular medium. For each experimental condition, values from three biological replicates (dots) with at least three technical replicates each are plotted. *B, C*. *P* values after applying GEE for percentage of depolarized cells among all groups are indicated as ** *P* <0.01.

Formatted: Font: Italic

Formatted: Not Superscript/ Subscript

Formatted: Font: Italic

Formatted: Font: 12 pt

Formatted: Font: 12 pt

Formatted: Font: 12 pt

~~Condition~~ Condition	~~KCl 15 mM~~ KCl 15 mM	~~KCl 60 mM~~ KCl 60 mM	~~KCl 300 mM~~ KCl 300 mM
KCl 0 mM (Control)	<0.001 ~~0.435~~ 0.435 ~~1.392~~ 1.392	<0.001 ~~0.900~~ 0.900 ~~1.899~~ 1.899	<0.001 ~~1.284~~ 1.284 ~~2.363~~ 2.363
KCl 15 mM		~~<0.001~~ <0.001 ~~1.592~~ 1.592 ~~1.365~~ 1.365	~~<0.001~~ <0.001 ~~0.849~~ 0.849 ~~1.698~~ 1.698
KCl 60 mM			~~<0.001~~ <0.001 ~~0.384~~ 0.384 ~~1.244~~ 1.244

Formatted: English (United States)

Formatted: English (United States)

Formatted: English (United States)

Formatted: English (United States)

SUPPLEMENTARY TABLES

Suppl. Table 1. Statistical generalized estimating equations (GEE) results for DiBAC validation assay in *B. subtilis.*

Legend:
P value
Coefficient
Relative Risk

Results for GEE analysis of the influence of [KCl] on the depolarization capacity of *B. subtilis* cells, considering the biological replicate as a grouping variable. *P* values correspond to the existence of significant difference between the KCl concentrations represented in row and column. The Relative Risk (*RR*) is based on the lower concentration value and estimates the change when moving to the second condition.

Suppl. Table 2: Statistical generalized estimating equations (GEE) results for the study of the bioelectrical analysis in *B. subtilis* growth dynamics.

Formatted: Font: Not Italic

Time	5 h<br>r_t = 0.00738	7 h<br>r_t = 0.00394
**3 h**<br>r_t = 0.01087	**0.032**<br>**0.117**<br>**1.074**	**<0.001**<br>**0.812**<br>**1.530**
**5 h**<br>r_t = 0.00738		**<0.001**<br>**0.695**<br>**1.425**

Legend:
P value
Coefficient
Relative Risk

Results for GEE analysis of the influence of time on the depolarization capacity of *B. subtilis* cells, considering the biological replicate as a grouping variable. *P* values correspond to the existence of significant difference between the different times represented in row and column. The Relative Risk (*RR*) is based on the lower time value and estimates the change when moving to the second condition.

Formatted: Font: Not Italic

Suppl. Table 3: Statistical generalized estimating equations (GEE) results for the study of the effect of neurotransmitters on *B. subtilis* cells.

Formatted: Font: Not Italic

Treatment (=Level)	Glutamate	GABA
**Control**	**<0.001**<br>**-0.884**<br>**0.583**	**<0.001**<br>**-1.238**<br>**0.448**
**Glutamate**		**<0.001**<br>**0.884**<br>**0.768**

Legend:
P value
Coefficient
Relative Risk

Results for GEE analysis of the influence of time of the presence of neurotransmitters on depolarization capacity of *B. subtilis* cells, considering the biological replicate as a grouping variable. *P* values correspond to the existence of significant difference between the different treatments (Control, Glutamate and GABA; also named as *factor levels*) represented in row and column. The Relative Risk (*RR*) is based on the control value and estimates the change when moving to the second treatment.

Formatted: Font: Italic

Suppl. Table 4: Statistical generalized estimating equations (GEE) results for DiBAC validation assay in *L. reuteri*.

Condition	KCl 15 mM	KCl 60 mM	KCl 300 mM

Legend:
P value
Coefficient
Relative Risk

**KCl 0 mM (Control)**	**<0.001**<br>**0.393**<br>**1.344**	**<0.001**<br>**0.638**<br>**1.593**	**<0.001**<br>**0.836**<br>**1.808**
**KCl 15 mM**		**<0.001**<br>**0.245**<br>**1.185**	**<0.001**<br>**0.443**<br>**1.345**
**KCl 60 mM**			**<0.001**<br>**0.197**<br>**1.135**

Results for GEE analysis of the influence of [KCl] on the depolarization capacity of *L. reuteri* cells, considering the biological replicate as a grouping variable. *P* values correspond to the existence of significant difference between the KCl concentrations represented in row and column. The Relative Risk (*RR*) is based on the lower concentration value and estimates the change when moving to the second condition.

Suppl. Table 5: Statistical generalized estimating equations (GEE) results for the study of the effect of neurotransmitters *on L. reuteri* cells.

Treatment (=Level)	Glutamate	GABA
**Control**	**<0.001**<br>**-0.483**<br>**0.724**	**<0.001**<br>**-0.487**<br>**0.722**
**Glutamate**		**0.933**<br>**-0.004**<br>**0.997**

Legend:
P value
Coefficient
Relative Risk

Results for GEE analysis of the influence of time of the presence of neurotransmitters on depolarization capacity of *L. reuteri* cells, considering the biological replicate as a grouping variable. *P* values correspond to the existence of significant difference between the different treatments (Control, Glutamate and GABA; also named as factor levels) represented in row and column. The Relative Risk (*RR*) is based on the control value and estimates the change when moving to the second treatment.

Formatted: Font: Italic

SUPPLEMENTARY INFORMATION

SPECIFIC GROWTH RATE or r_t

r_t is the specific growth rate, a parameter that we use as an indicator of the physiological state of the bacterial cells, and it is defined by the following equation:

$$r_t = 1 - \frac{OD_t}{k}$$

where r = growth rate

OD_t = Optical Density values at each measured time

k = estimated carrying capacity of the culture

r (0.0127) and k (1.078) are calculated by fitting OD data to a Verhulst growth curve, performed by a non-linear regression analysis on the following function:

$$y(t) = \frac{y_0 k \exp(rt)}{k + y_0(\exp(rt) - 1)}$$

where $y_0 = 0.014$ (estimated from OD_{600} data)

Nernst EQUATION FOR EQUILIBRIUM POTENTIAL(V_{Eq}; Eq.1)

$$V_{Eq.} = \frac{RT}{zF} ln\left(\frac{[K^+]_{out}}{[K^+]_{in}}\right) \quad (1)$$

where $V_{Eq.}$ is the equilibrium potential;
R is the universal gas constant and is equal to 8.314 $J.K^{-1}.mol^{-1}$ (Joules per Kelvin per mole);
T is the temperature in Kelvin (K = °C + 273.15);
Z is the valence of K^+ (+1; unitless);
F is the Faraday's constant and is equal to 96,485 $C.mol^{-1}$ (Coulombs per mole);
($[K^+]_{out}$)t is the concentration of K^+ in the extracellular medium (mM);
($[K^+]_{in}$)is the concentration of K^+ in the intracellular medium (mM).

Formatted: Not Highlight

Formatted: Not Highlight

Formatted: Not Highlight

Formatted: Not Highlight

Formatted: Not Highlight

Formatted: Not Highlight

Formatted: Not Highlight

Formatted: Not Highlight

Formatted: Not Highlight

Formatted: Not Highlight

Formatted: Not Highlight
